# Production of Plant-Derived Oleuropein Aglycone by a Combined Membrane Process and Evaluation of Its Breast Anticancer Properties

**DOI:** 10.3389/fbioe.2020.00908

**Published:** 2020-09-29

**Authors:** Rosalinda Mazzei, Emma Piacentini, Monica Nardi, Teresa Poerio, Fabio Bazzarelli, Antonio Procopio, Maria Luisa Di Gioia, Pietro Rizza, Rosangela Ceraldi, Catia Morelli, Lidietta Giorno, Michele Pellegrino

**Affiliations:** ^1^Institute on Membrane Technology, National Research Council, ITM-CNR, Rende, Italy; ^2^Department of Pharmacy, Health and Nutritional Sciences, University of Calabria, Rende, Italy

**Keywords:** oleuropein aglycone, membrane bioreactor, membrane emulsification, breast cancer, integrated membrane systems

## Abstract

Natural products and herbal therapies represent a thriving field of research, but methods for the production of plant-derived compounds with a significative biological activity by synthetic methods are required. Conventional commercial production by chemical synthesis or solvent extraction is not yet sustainable and economical because toxic solvents are used, the process involves many steps, and there is generally a low amount of the product produced, which is often mixed with other or similar by-products. For this reason, alternative, sustainable, greener, and more efficient processes are required. Membrane processes are recognized worldwide as green technologies since they promote waste minimization, material diversity, efficient separation, energy saving, process intensification, and integration. This article describes the production, characterization, and utilization of bioactive compounds derived from renewable waste material (olive leaves) as drug candidates in breast cancer (BC) treatment. In particular, an integrated membrane process [composed by a membrane bioreactor (MBR) and a membrane emulsification (ME) system] was developed to produce a purified non-commercially available phytotherapic compound: the oleuropein aglycone (OLA). This method achieves a 93% conversion of the substrate (oleuropein) and enables the extraction of the compound of interest with 90% efficiency in sustainable conditions. The bioderived compound exercised pro-apoptotic and antiproliferative activities against MDA-MB-231 and Tamoxifen-resistant MCF-7 (MCF-7/TR) cells, suggesting it as a potential agent for the treatment of breast cancer including hormonal resistance therapies.

## Introduction

Breast cancer (BC) is a disease that features several morphological aspects, different therapeutic responses, and variable clinical outcomes. In industrialized countries, it represents the most common cause of death in women (Ferlay et al., 2015; Kohler et al., 2015). Current therapeutic cancer treatments are based on various methods including surgical removal, radiotherapy, chemotherapy, biological therapy, and hormone therapy. The side effects of these therapies are innumerable, and some of them are related to the cytotoxic activity of drugs that act indistinctly both on cancer cells and on healthy ones. The severe side effects of these therapies, such as nausea, vomiting, diarrhea, mucositis, and myelosuppression decrease the quality of life of the patients (Sharma et al., 2005). Moreover, increasing instances of tumors with resistance to current therapeutic compounds has become a problematic issue, which has pushed research toward new anticancer agents (Vidal et al., 2014; Carnero et al., 2016).

In the last few decades, vegetal products have been recognized as effective and economic sources of new synthetic therapeutic compounds and many scientists have started to focus their research on this sector (Cragg and Newman, 2013; Omogbadegun, 2013). One of the main strategies adopted by these researchers addresses the need to find synthetic routes for these vegetal products using sustainable technologies (Woodley, 2008; Abdelraheem et al., 2019).

Membrane-based technologies and in particular membrane bioreactors (MBRs) (Mazzei et al., 2009, 2010a, 2013; Giorno et al., 2013; Macedonio and Drioli, 2017) are widely recognized as more ecological processes of engineering since they fulfill the different criteria which make them more “green,” such as equipment size, energy consumption, process flexibility, and so forth. MBRs are also widely investigated in the pharmaceutical application for the production of different drugs (Mazzei et al., 2010b, Mazzei et al., 2017; Piacentini et al., 2017).

In our previous works, the oleuropein aglycone (OLA) was produced using different MBRs systems. OLA is a vegetal product with many therapeutic applications due to its antihyperglycemic, anti-Alzheimer disease, anti-inflammatory, antioxidant, and other properties (Galiano and Villalain, 2015). In particular, it was produced by the enzymatic (β-glucosidase) hydrolysis of oleuropein (OLE) extracted from olive leaves (Mazzei et al., 2010b, 2012; Ranieri et al., 2018; Piacentini et al., 2019). In the above-mentioned MBRs, the enzyme was immobilized within the membrane and the maximum conversion achieved was 60%. As a consequence, the recovered OLA was not purified from the substrate, since unconverted OLE was also present in the collected solution. In the batch system, the co-product glucose also promotes a competitive inhibition, as already reported in Ranieri et al. (2018).

**FIGURE 1:**
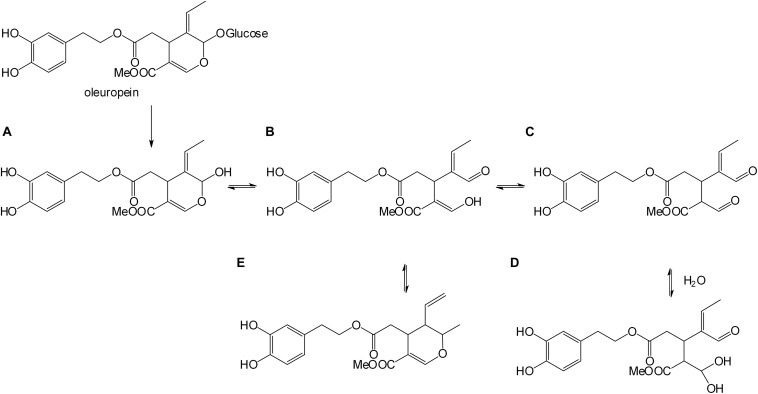
Enzymatic oleuropein hydrolysis. Aglyconic product **(A)**, monoaldehyde **(B)**, dialdehyde **(C)**, geminal diol **(D)**, and dihydropyran **(E)** forms.

For OLA formation occurring after β-glucosidase action (Scheme in Figure 1), the aglyconic product (A) is highly unstable and rearranges in different isomers (De Nino et al., 2000; Di Donna et al., 2011). It undergoes an isomerization process that leads to the formation of monoaldehyde (B), dialdehyde (C), geminal diol (D), and dihydropyran (E) characterized by ionspray ionization tandem mass spectrometry (IS-MS/MS) (De Nino et al., 2000), and quantified by ultra-high-performance liquid chromatography-electrospray ionization-tandem mass spectrometry (UHPLC-ESI-MS/MS) (Di Donna et al., 2011).

In this study, an MBR was specifically designed to minimize product inhibition during the enzymatic reaction, to obtain the final solution purified OLA. The OLE used in this work was produced by a sustainable method starting from olive leaves (Procopio et al., 2009). In the MBR, the reaction was carried out in a stirred tank reactor (STR), using an excess of the enzyme, then the solution was sent to an ultrafiltration (UF) process, which permitted enzyme recovery for re-use and product removal. The UF permeate was then sent to a membrane emulsification (ME) process, to promote the extraction of the compound of interest in a volatile green organic solvent. The subsequent evaporation of the solvent permitted the recovery of dry OLA. The overall proposed strategy provides a possible method that can be used to (bio) synthesize OLA from renewable waste material, representing an attractive productive alternative to its isolation from olive oil.

Several studies (*in vitro* and *in vivo*) have reported the antiproliferative and proapoptotic effects of OLE (Sirianni et al., 2010; Casaburi et al., 2013; Barbaro et al., 2014; Abtin et al., 2018; Antognelli et al., 2019; Bayat et al., 2019; Liu et al., 2019). Particularly, it has been demonstrated that OLE can show apoptotic effects in a human MCF-7 cell line (Han et al., 2009). However, there is a common consensus that OLA could have a higher therapeutic effect than OLE, due to its higher hydrophobic character, which allows interaction directly with biological membranes (Galiano and Villalain, 2015), increasing its local concentration (Leri et al., 2016) and its beneficial pharmacological effect on cells.

Oleuropein aglycone is not yet commercially available as a pure compound and just a few studies have reported the potential of the compound on BC treatment (Impellizzeri et al., 2011; Pantano et al., 2017; Xu et al., 2018), when in combination with the other polyphenols extracted from extra virgin olive oil (EVOO) (Menendez et al., 2008) or using the single compound on MCF-7, SKBR3, and MCF-7/(pBABE)HER2 cells. Among the different polyphenols from EVOO (tyrosol, hydroxytyrosol), OLA, which was separated from the other polyphenols by a semi-preparative reverse-phase high-performance liquid chromatography, showed the best performance in decreasing BC cell viability (Menendez et al., 2007). The mentioned article highlighted the higher anticancer properties of OLA than OLE toward BC cell lines. This work also proposed that the higher anticancer effect was attributed to the higher lipophilicity of the OLA than OLE, which permits improved incorporation in the cell membrane as well as better interaction with other lipids.

A sustainable production process for OLA must be studied to replace the expensive existing method of olive oil extraction, without altering its anticancer properties. Taking this into account, this is the first study to assess the biological activity of OLA produced by the integrated membrane process toward two different very aggressive BC cell lines, intending to verify if the production process could alter the anticancer properties of this important phytotherapic compound. In particular, the potential cytotoxic effects of this bioderived compound on MDA-MB-231 triple-negative and MCF-7 tamoxifen resistant (MCF-7/TR) BC cell lines have been studied, to establish its possible mechanism of action.

The MDA-MB-231 cell line is a highly aggressive, invasive, and poorly differentiated triple-negative breast cancer (TNBC) cell line as it lacks estrogen receptor (ER) and progesterone receptor (PR) expression, as well as HER2 (human epidermal growth factor receptor 2) amplification. The MCF-7/TR is a tamoxifen-resistant cell line derived from the selection of parental ERα+ MCF- 7 cells. The pro-apoptotic and antiproliferative activities of OLA against BC cells, regardless of ER status, have been demonstrated.

## Materials and Methods

### Chemicals

β-glucosidase from almond (Cod. G4511) and the ethyl acetate were supplied by Sigma-Aldrich. Solvents for HPLC, NMR, LC-QTOF-MS (acetonitrile, methanol, dichloromethane, dimethyl sulfoxide, deuterated chloroform) were supplied by Carlo Erba and Sigma-Aldrich. NaH_2_PO_4_ and Na_2_HPO_4_, supplied by VWR, were used to prepare phosphate buffer (50 mM, pH 6.5) for enzymatic and substrate solutions. Bicinchoninic acid kit (BCA) was used to measure protein concentration, supplied by Sigma-Aldrich.

### HPLC Analysis

The HPLC (Hewlett–Packard 2100) was equipped with a Rheodyne injector, a 20 μl loop, and a UV detector (280 nm) (Agilent Technologies Hewlett-Packard-Strasse 8, 76337, Waldbronn, Germany). The column used is a Phenomenex Jupiter C18 (250–4.6 mm, 5 μm) heated to 30°C with an elution gradient from 100% of H_2_O (5 min) to 35% of CH_3_CN (over 10 min), followed by 35–90% of CH_3_CN (5 min). The concentration of OLE and OLA in the samples coming from the integrated membrane process was measured by HPLC, by the method reported in Mazzei et al., 2012. A reverse silica C18 column (250–4.6 mm, 5 μm, Grace, Adsorbosphere XL) was used. A mixture of CH_3_CN/H_2_O (21:79) acidified with o-phosphoric acid (up to pH 3) was used as a mobile phase, injecting 5 μl of sample volume and setting the UV detector at 280 nm.

### LC-QTOF-MS Analysis

An Agilent 6540 UHD Accurate–Mass LC-Q-TOF-MS (Agilent, Santa Clara, CA, United States) equipped with an Electrospray ionization source (Dual AJS ESI) operating in positive ion mode was used to carry out the analyses of ultra-high-pressure liquid chromatography (UPLC) combined with quadrupole time-of-flight mass spectrometry (Q-TOF-MS). We used a C18RP analytical column (Poroshell 120, SB-C18, 50 × 2.1 mm, 2.7 μm) heated to 30°C. For the analysis, we used an elution gradient from 5 to 95% of CH3CN [0.1% formic acid (FA) over 13 min] in an aqueous solution (0.1% FA), with a flow rate of 0.4 ml/min.

### NMR Spectra

^1^H-NMR and ^13^C-NMR spectra were recorded at 300 MHz, and at 75 MHz, respectively, using a Bruker WM 300 system. The samples were solubilized in DMSO for OLE analysis or in CDCl_3_.for OLA analysis. Chemical shifts are given in parts per million (ppm) using tetramethylsilane as the internal (0.0 ppm). Coupling constants (J) are given in hertz.

### Microwave-Assisted Extraction of Oleuropein From Olive Leaves

Coratina cultivar of Olea europaea leaves, supplied by “Consiglio per la ricerca in agricoltura e l’analisi dell’economia agraria (CREA),” were used as initial feedstock for oleuropein extraction. The sample was first dried at 50°C for 48 h, pulverized, and then kept at room temperature until use. One hundred grams of the obtained sample was suspended in water (800 ml) and placed in a Pyrex round-bottom flask (equipped with a jacketed coiled condenser) in a domestic microwave oven. After 10 min at 800 W, samples were filtered and the water solution was dried under reduced pressure by Heidolph Rotary Evaporator, Laborota 4000. The obtained dried sample was then washed with acetone and filtered and the solution was evaporated under reduced pressure. Pure oleuropein was obtained by purifying the crude product by liquid chromatography on a Supelco VersaFlash station. A silica cartridge and a mixture of CH_2_Cl_2_/MeOH 8:2 as a mobile phase were used. The purified sample was analyzed by LC-Q-TOF-MS. By this method, a yield of pure oleuropein of 5% was obtained.

### Combined Membrane Process to Produce Dry OLA

**FIGURE 2:**
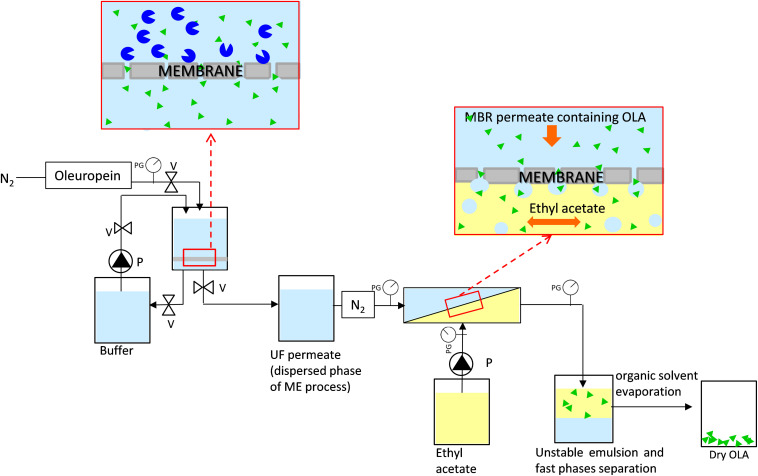
Membrane system used to produce and extract oleuropein aglycone composed by a membrane bioreactor and a membrane emulsification process, PG, pressure gauges; V, valves; P, pump.

The integrated system to produce the dry OLA is composed of an MBR and a ME process (Figure 2). The MBR is composed of a stirred tank (ST) (50 mL) in which the enzymatic reaction occurred (37°) and a flat ultrafiltration membrane (30 kDa, regenerated cellulose membrane, diameter 47 mm, Millipore) to separate the produced OLA from the enzyme. Inlet and outlet pressure were measured by digital pressure gauges (Keller). A thermostated jacket was used to thermally control (37°) the MBR unit. The oleuropein powder and enzyme were dissolved in 50 mM phosphate buffer (pH 6.5). The initial oleuropein solution concentration is 2.5 mM, while the enzyme concentration is 3.8⋅10^–3^ mM (10 mg).

After each reaction cycle in the STR, the solution was ultrafiltered (under nitrogen pressure) to separate the product and retain the enzyme (applied transmembrane pressure: 0.2 bar) and a new volume of substrate (30 mL) was added into the reactor using a peristaltic pump (Masterflex), to start the subsequent reaction cycle. The reaction solution was not completely ultrafiltered (10 mL remained as retentate). To remove the glucose present in the remaining retentate, washing steps using phosphate buffer (using 50 mM phosphate buffer pH 6.5) were carried out, adding 30 mL of buffer to the remaining retentate (10 mL) and ultrafiltrating the solution at 0.2 bar.

Some experiments (e.g., optimization of enzyme concentration, etc.) in a STR were also performed to study the effect of the competitive inhibition of the glucose. The operating conditions used for the batch STR were the same as the conditions used in the STR in the MBR.

The permeate, coming from every reaction cycle of the MBR and containing the purified OLA, was used as a dispersed phase for the ME process (Figure 2) and it was pressed under nitrogen gas (flux = 48 ± 8 Lh^–1^ m^–2^) through the membrane. For this process, a hydrophobic SPG (Shirasu porous glass, SPG Technology Co., Ltd., Japan) membrane, with tubular configuration (0.4 μm, 8.7 mm inner diameter, 0.65 mm wall thickness, 31.3 cm2) was used. Ethyl acetate was used as the continuous phase and it was flushed, along the lumen side of the membrane, under pulsed flow, by a programmable peristaltic pump (Digi-Staltic double-Y Masterflex pump Micropump, model GJN23.JF1SAB1) (shear stress = 1.34 Pa). Aqueous droplets are formed at the pore outlet when the dispersed phase, containing OLA, meets the organic phase at the membrane surface. Owing to this process, OLA is extracted into the organic phase at the water-ethyl acetate interface. Due to the non-stabilized droplets of the formed emulsion (Piacentini et al., 2019), fast separation of the two phases was promoted, which guaranteed an easy recovery of the phase of interest. Dry OLA was produced at the end of the process by ethyl acetate evaporation.

The conversion (%) in the STR and the MBR was measured as the ratio between the mass of the OLA produced at the end of the cycle respect to the initial substrate mass.

The Extraction efficiency (EE) of OLA has been determined according to the following equation:

$$EE=\frac{C_{EA}V_{EA}}{C_{H_{2}O}V_{H_{2}O}}$$

Where CEA and VEA are OLA concentration in ethyl acetate and the volume of ethyl acetate were used as the continuous phase, respectively, while C_H_2_O_ and V_H_2_O_ are OLA concentration in water and the volume of water were used as the dispersed phase, respectively.

### Generation of MCF-7 Tamoxifen Resistant Cell Line (MCF-7/TR)

The MCF-7/TR cell line was selected after the long-term cultivation of parental ERα+ MCF- 7 cells. To induce resistance to tamoxifen, cells were exposed stepwise against increasing concentrations of 4-hydroxytamoxifen (4-OHT, Sigma-Aldrich, Italy), starting from 10^–9^M up to a final concentration of 10^–6^M. Cells were re-fed with a fresh growth medium containing the drug every 2–3 days. The acquired resistance to the antiestrogen was checked on a scheduled basis by the lack of any inhibitory effect on the proliferation of resistant cell lines compared to the parental ones (Supplementary Figures S1A,B, Supporting information).

### Cell Cultures

MCF-7 and MDA-MB-231 BC cell lines and MCF10A (normal epithelial cells) were stored and used according to the supplier instructions. In all the solution fetal bovine serum (5%), l-Glutamine (1%), and penicillin-streptomycin (1 mg/ml) were present.

Cell lines were acquired and authenticated from Interlab Cell Line Collection (ICLC, Genova, Italy), they were tested monthly for negativity to *Mycoplasma* (MycoAlert, Lonza) and they were maintained as previously described (Pellegrino et al., 2018).

### Cell Viability Assay (SRB)

A sulphorhodamine B assay (SRB) was used to evaluate the total biomass by staining cellular protein content. After treatment with vehicle (−) or OLA at 50 and 100 μM for 24, 48, and 72 h in 96-well plates, 10% trichloroacetic acid (TCA) for 1 h at 4°C was used to fix the cells. After fixation, SRB was used for staining (15 min) and the cells were washed 3 times with 1% acetic acid. Finally, the absorbance at 540 nm was measured by spectrophotometric analysis. GraphPad Prism 4 (GraphPad Software, Inc., San Diego, CA, United States) was used to calculate IC50 values.

### Clonogenic Assay

Cells were plated in growth medium (GM) in 6-multiwell plates. The next day, they were treated with vehicle (−) or OLA at 50 and 100 μM. OLA treatment was renewed every 3 days. After 14 days, surviving colonies were stained with Crystal Violet and the images were acquired by Olympus BX51 inverted microscope. The obtained data by the Image J software were elaborated and normalized.

### Soft Agar Anchorage-Independent Growth Assays

Cells were plated as previously described (Pellegrino et al., 2018). After 2 days, vehicle (−) or treatments (OLA at 50 and 100 μM) were added and replaced every 3 days. After 21 days, 3-(4,5-dimetiltiazol-2-il)-2,5-difeniltetrazolio (MTT) was added to the plate and it was incubated for 4 h at 37°C. After overnight incubation at 4°C, colonies >50 μm diameter from triplicate assays were counted using an Olympus BX51 microscope.

### Flow Cytometry

Cells after plating were treated with vehicle (−) or OLA at 50 and 100 μM for 24 h. The cells were harvested by trypsinization and resuspended in propidium iodide solution after treatment with RNase as described above (Rizza et al., 2016). FACScan flow cytometer (BD, United States) was used for measuring the DNA content was used and the data were acquired using CellQuest software. ModFit software was then used to determine the cell cycle profile was used.

### Reverse Transcription and Real Time PCR (qRT-PCR)

After plating and starvation, the cells were treated with vehicle (−) or OLA at 50 and 100 μM for 12 and 24 h. TRIZOL reagent (Life Technologies, Italy) was used according to the manufacturer’s instructions for total RNA extraction. Total RNA (2 μg) was reverse transcribed with RETRO script kit (Ambion, Life Technologies, Italy). cDNA (diluted 1:3) was analyzed in the iCycler iQ^TM^ detection system (Bio-Rad) in triplicate, using SYBR green Master Mix (from Bio-Rad). Each sample was normalized vs. GAPDH mRNA content. All gene primer sequences including Cyclin D1, Cyclin E, p21cip1/waf1, p53, p27Kip1, Bcl-2, Bcl-XL, Bax, Bad, and GAPDH are shown in Supplementary Table S1 in the Supplementary Information. The results were calculated and expressed as previously reported (Catalano et al., 2007).

### Western Blot (WB) Analysis

Equal amounts of proteins were resolved on SDS-page gel, as previously described (De Amicis et al., 2016). WB images are indicative of at least 3 different experiments.

Cyclin D1 (sc-718), cyclin E (sc-481), p21cip1/waf1 (p21) (sc-756), p53 (sc-126), p27Kip1 (p27) (sc-53871), Bcl-2 (sc-7382), Bcl-XL (sc-8392), Bax (sc-7480), Bad (sc-8044), and β-actin (sc-69879) were provided by Santa Cruz Biotechnology, Inc., Dallas, TX, United States, while PARP (#9532), Caspase3 (#9665), and Caspase8 (#9746) were purchased from Cell Signaling Technology, Netherlands, EU.

### Statistical Analysis

ANOVA and Newman-Keuls’ testing were used to determine differences in means for statistical analysis. The band intensities in WBs represent the optical density and express the percentage vs. control. Data are shown as the mean ± SD of three different experiments, each performed in triplicate ^∗^ *p* ≤ 0.05 vs. control.

## Results and Discussion

### Production of Oleuropein Aglycone by Integrated Membrane Process

In this study, the glucose inhibition on the biocatalyst was controlled by adding a higher enzyme amount (enzyme: 1.5 10^–7^ mol, substrate: 7.5 10^–5^ mol, 2.5 mM) compared to the one previously reported (Ranieri et al., 2018), which achieved a conversion of about 93% in about 5 h in the STR. The same strategy was also used for other enzymatic systems in the presence of competitive inhibition (Andrić et al., 2010). However, the high price of the enzyme and the need for a subsequent product purification phase limited the development of this system on a large scale, therefore the recovery of the enzyme employing a membrane process is strongly recommended. To improve the conversion and purity of OLA, its production was carried out in an MBR consisting of an STR coupled with a UF process. To control the competitive inhibition caused by the co-product glucose (Ranieri et al., 2018), we studied the effect of glucose amount in contact with the enzyme, and its removal by UF (Table 1).

**TABLE 1:** Operating conditions used in the MBR and OLA conversion in the different reaction cycles in the MBR.

SERIE	Operative conditions				Conversion%
	Reaction cycles in the MBR	Time contact between Enzyme/glucose (h)	Amount of glucose in contact with the enzyme prior to the substrate addition (mol)	Ultrafiltration with buffer to remove glucose in solution after UF process	
1	1	5			93
	2		2.43⋅10^–5^**		82
	3		4.60⋅10^–5^**		23
2	1		1.50 10^–7^*	x	96
	2			x	93
	3			x	94

* Moles of glucose bound to the enzyme, assuming that the glucose moles are equal to enzyme moles. ** Moles of glucose remained in solution.

After each reaction cycle, the reaction mixture was not completely ultrafiltered (10 mL of retentate remained into the tank) to recover the enzyme. The use of this procedure, however, does not permit the complete removal of the glucose, causing competitive inhibition at the beginning of the next reaction cycle. This was confirmed (Table 1) by the continuous decrease of oleuropein conversion obtained in SERIE 1. Indeed, between the first and the second reaction cycle, the moles of glucose remained in contact with the enzyme were 2.43 10^–5^ and consequently, we observed a decrease in the conversion of about 10% (Table 1). The moles of glucose, which were present before the third cycle, are almost doubled (4.6⋅10^–5^ moles) and this causes a significant drop in the degree of conversion (only 22%). To avoid this competitive inhibition, a washing step (with a buffer solution) between the cycles was added (Table 1, SERIE 2) This procedure combined with the new substrate addition allows maintaining the maximum conversion obtained in the first cycle and to overcome the inhibition of the glucose linked to the enzyme since the enzyme has more affinity for the substrate than the inhibitor (K_M_: 1.17 mM, K_i_: 1.93 mM, Ranieri et al., 2018). After the washing step (Table 1, SERIE 2) the glucose remained in the retentate solution is washed out, but not the enzyme that thanks to membrane process is retained and can be reused for the next reaction cycle.

**FIGURE 3:**
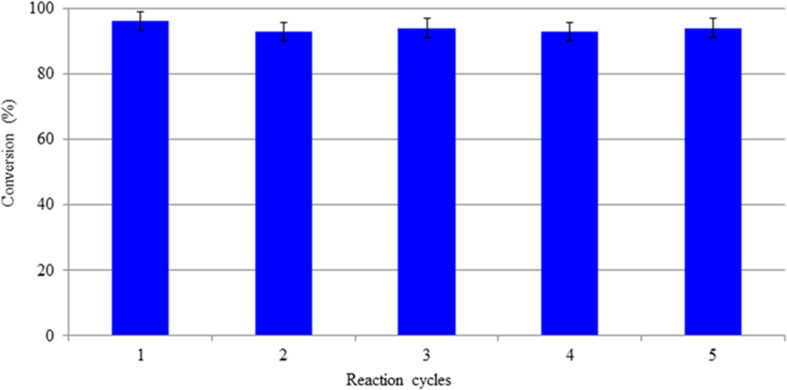
Conversion of OLE in the MBR during different reaction cycles.

As a result, it was possible to carry out five reaction cycles with no decay in conversion (Figure 3).

The optimized MBR was then integrated with a ME process to achieve the extraction of OLA in the ethyl acetate as reported in Piacentini et al. (2019). When the dispersed phase, containing OLA coming from the MBR permeated through the membrane of the ME system, water droplets are generated at the membrane pore level that quickly separated from the continuous organic phases. The high surface area/volume ratio of the produced droplets allowed the continuous and high extraction (90%) of OLA in ethyl acetate under mild (e.g., low shear stress) operating conditions, from which the compound of interest could be easily recovered after solvent evaporation. The optimized integrated membrane process and in particular the strategy to decrease the inhibition effect of the glucose during the reaction, permitted to obtain a high purity of OLA in mild operative conditions. Besides, the enzyme which is one of the most expensive compounds in productive systems can be re-used for different reaction cycles with no decay in the activity.

### Characterization of Obtained Oleuropein Aglycone

To check that the integrated membrane process had not altered the phytotherapic compound, the OLA produced by HPLC, H1-NMR, and LC-MS were characterized. The H1-NMR spectrum (Supporting information) in CDC_*l*__3_ showed the aldehyde proton signals of the isomeric forms of OLA in the range 9.10–9.95 ppm. In particular, the signal of the monodialdehydic proton of the form D (scheme in Figure 1) (9.45 ppm), two aldehyde proton signals of the dialdehydic form C (9.52 and 9.60 ppm), and the aldehyde proton signal of the monodialdehydic form B (9.80 ppm). The signals (Supporting information) of hydrate forms B and D are less intense than the more lipophilic forms C and E.

The HPLC spectrum showed five signals reported to the forms A, B, C, D, and E (see Supplementary Figure S4). These five forms were detected using an elution gradient from 100% of H_2_O 5 min to 35% of CH_3_CN over 10 min, followed by 35–90% of B in 5 min.

**FIGURE 4:**
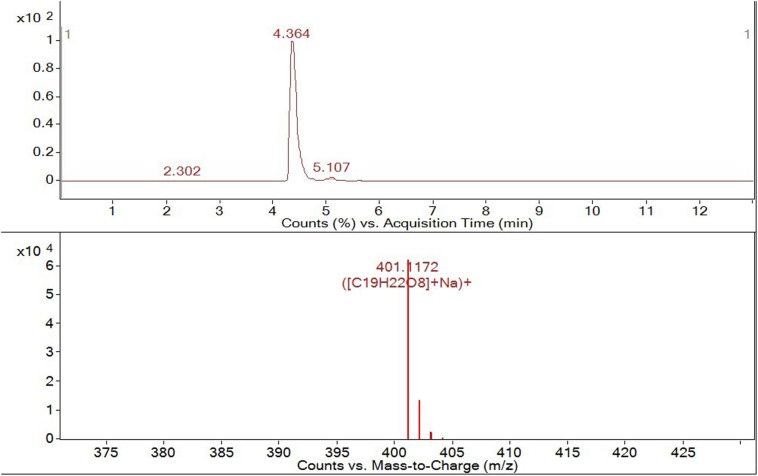
HPLC-ESI-QTOF-MS tr = 4.364 min m/z 401. 1172[M + Na^+^].

LC-MS analysis (Figure 4) confirmed the presence of OLA isomers with an LC retention time of 4.364 min and an MS molecular ion peak of m/z = 401. 1172 [M + Na]^+^.

The presence of all OLA isomers confirmed the suitability of the integrated membrane process for the production of this compound since we observed no modifications in the molecule structure after its production and extraction.

### Oleuropein Aglycone Inhibits Breast Cancer Cell (BCC) Growth

**FIGURE 5:**
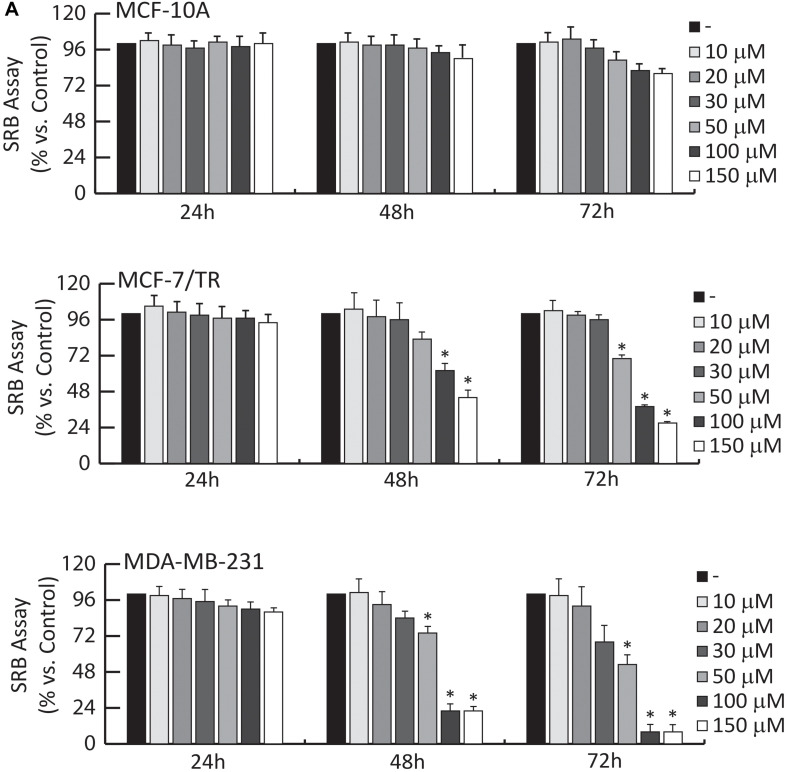
Effects of OLA compound on breast cancer growth. **(A)** SRB growth assays. MCF-10A, MCF-7/TR, and MDA-MB-231 cells treated with vehicle (−) or increasing doses (1-10-20-30-50-100-150 μM) of OLA for 24-48-72 h, as indicated. The results are expressed as % respect to control cells (−). ±S.D. relative to vehicle-treated cells and is representative of three different experiments each performed in triplicate ^∗^*P* < 0.05 treated vs vehicle-treated cells (−).

The OLA produced by the integrated membrane process was subsequently used to evaluate the effect on BC viability (Figure 5) using MDA-MB-231 triple-negative and ER-α+ Tamoxifen resistant (MCF-7/TR) BCC.

The effects of different doses (1, 10, 20, 30, 50, 100, and 150 μM) of OLA were tested, using SRB assays on cell viability. Among the different doses, results demonstrated that 50 and 100 μM significantly inhibited cell viability in MDA-MB-231 and MCF-7/TR BC cells (Figure 5A).

The IC50 (half-maximal inhibitory concentration) values for OLA are reported in Table 2. In normal breast epithelial cells (MCF-10A), the treatment with this compound elicits no considerable effects on cell viability (Figure 5A). Next, we evaluated: (1) the anti-proliferative effects induced by OLA on anchorage-dependent conditions (clonogenic assay, Supplementary Figure S6A); (2) the anchorage-independent BCC growth (soft agar assays) that mimic *in vivo* biologic features of tumors (Supplementary Figure S6B). According to the SRB assays OLA treatments, in a dose-dependent manner, significantly reduced colony formation in BCC tested (Supplementary Figures S6A,B).

**TABLE 2:** IC_50_ values of OLA for BCC.

Cell lines	IC_50_ (μM)	95% confidential interval
MCF-7/TR	70	13–18.2
MDA-MB-231	53	15.5–28.7

*IC_50_ half-maximal inhibitory concentration.*

In summary, these results suggest the capability of OLA to inhibit cell growth and proliferation in a selective manner on BCCs, without affecting normal breast epithelial cells (MCF-10A). Interestingly, OLA can exert an antiproliferative effect in Tamoxifen resistance conditions (MCF-7/TR), suggesting its use as an adjuvant strategy in the treatment of patients, which are refractory to hormonal therapies.

### Effects of Oleuropein Aglycone on Cell Cycle in BCCs

**FIGURE 6:**
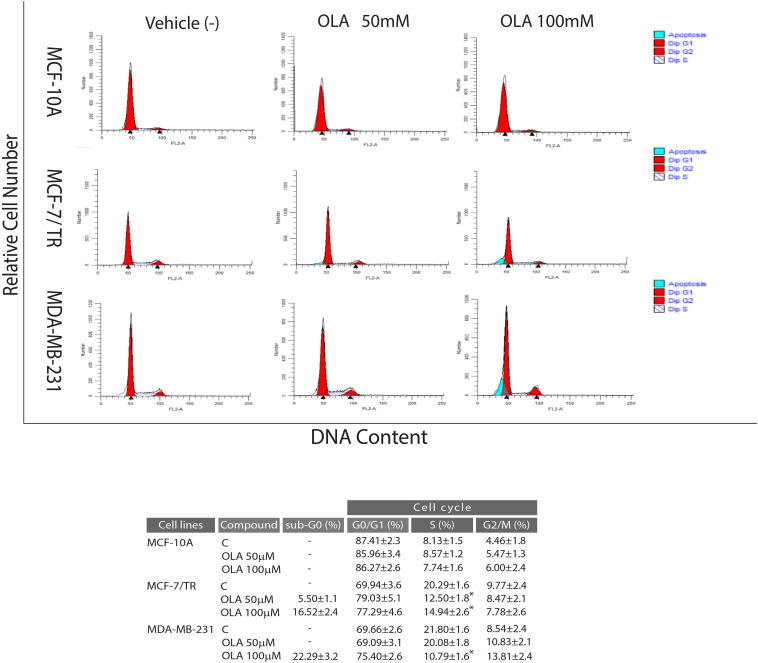
Effects of OLA compound on cell cycle distribution in breast cancer cells. MCF-7/TR cells, MDA-MB-231 cells, and MCF-10A were treated with vehicle (−) and 50 or 100 μM of OLA compounds for 24 h, stained with propidium iodide (PI) and analyzed on a FACScan flow cytometer. Quantitative analysis of percentage gated cells at G0/G1, S, G2/M, and sub-G0 phases are shown. ^∗^*P* < 0.05 OLA treated vs vehicle-treated cells (−).

To verify whether the inhibition of cell growth induced by the OLA compound was a perturbation of cell-cycle, Flow Cytometric cell-cycle analysis in all BCC after 24 h of treatment with vehicle (−) or OLA at 50 and 100 μM were performed. As shown in Figure 6A, OLA treatment induced a cell cycle arrest in the G0/G1 phase and a reduction of the fraction of cells in S-phase, as well as an increased % of apoptotic cells in both BCC used. In MCF-10A, as expected, OLA at 50 and 100 μM did not elicit any effects on the cell cycle (Figure 6).

In apoptotic pathways, the activation axis including p21Cip1/WAF1, p53, p27, cyclin D1, and cyclin E are implicated in tumor pathogenesis and development of BC as well as in the resistance to anticancer therapy. In this context, the capability of a substance to interfere with this signaling could represent a promising option in investigating potential treatments for BC (Lim et al., 2015).

**FIGURE 7:**
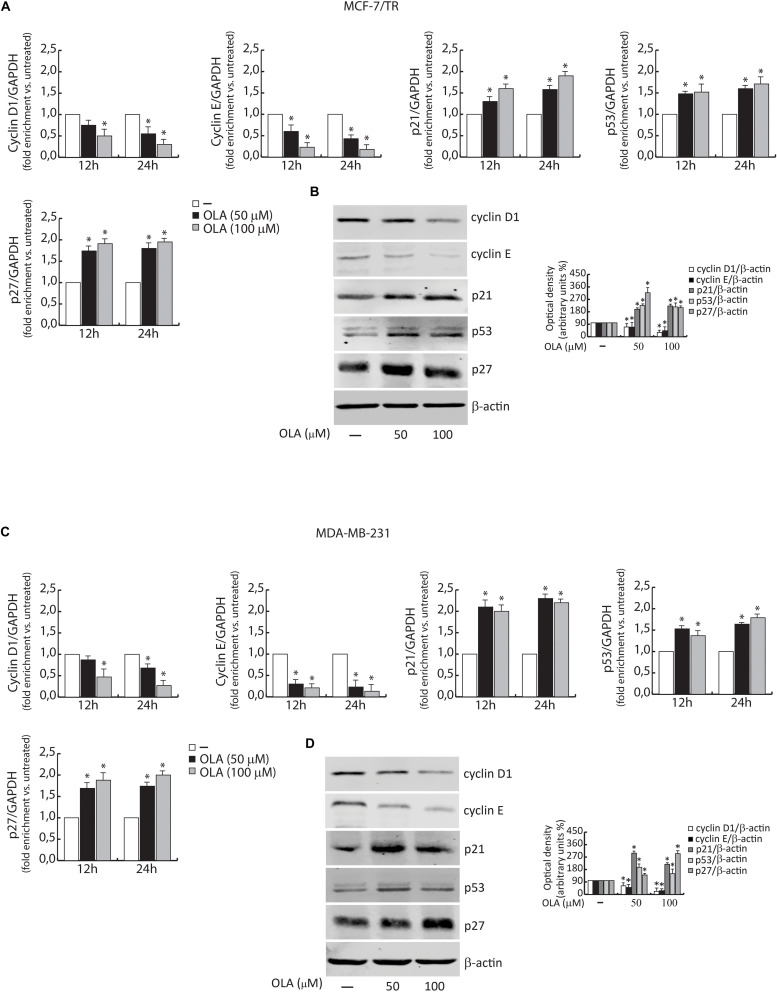
Effect of OLA compound on mRNA **(A,C)** and protein expression **(B,D)** in MCF-7/TR and MDA-MB-231 cells. **(A,C)** BCC was treated with vehicle (−) and 50 or 100 μM of OLA compound for 12 and 24 h before lysis. Cyclin D1 and E, p21^Cip1/WAF1^, p53, and p27 mRNA content, evaluated by real-time RT-PCR. Each sample was normalized to its GAPDH mRNA content. The values represent the means ± S.D. of three different experiments, each performed in triplicate. **(B,D)** Equal amounts of total cellular protein extracts were analyzed for Cyclin D1 and E, p21^Cip1/WAF1^, p53, and p27 and protein levels by immunoblotting analysis. B -actin was used as the loading control. The histograms represent the mean ± SD of three separate experiments in which band intensities were evaluated in terms of optical density arbitrary units (OD) and expressed as a percentage of control which was assumed to be 100%. ^∗^*P* < 0.05 OLA treated vs vehicle-treated cells (−).

In order to elucidate the molecular mechanism through which the OLA compound induced the anti-proliferative effects and the capability to interfere in the pathways previously mentioned, the levels of mRNA and proteins associated with cell cycle-regulation were also examined (Figure 7).

Cells were exposed to the OLA compound at 50 and 100 μM for 12–24 and 48 h and the whole-cell extract were subjected to RT-PCR or immunoblotting analysis, respectively.

Similar to the results obtained on the cell cycle, these results indicated that a significant down-regulation of cyclin D1 and cyclin E expression in both BCC was observed. In the same experimental conditions, an up-regulation of p21Cip1/WAF1, p53, and p27 was evidenced (Figures 7A–D). These results confirm our hypothesis about the capability of OLA to interfere with the signaling, suggesting its apoptotic role in BCCs, including the tamoxifen resistant cells.

### Oleuropein Aglycone Induces Apoptosis in Breast Cancer Cells

**FIGURE 8:**
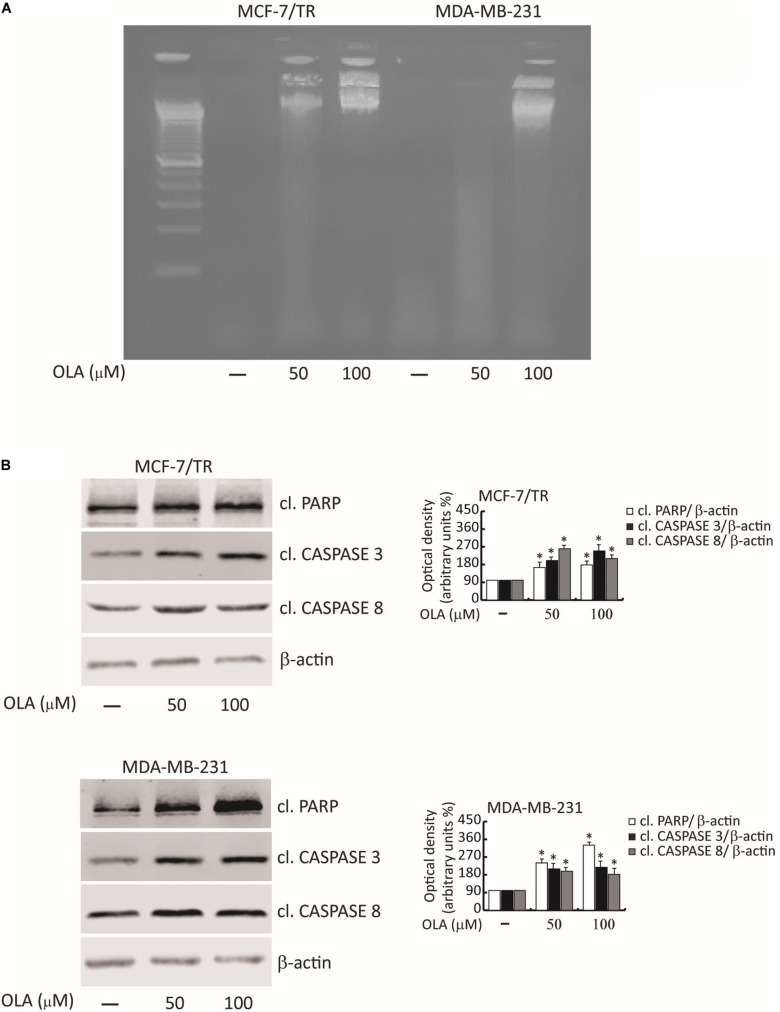
OLA triggers apoptotic cell death in breast cancer cells. **(A)** DNA laddering performed in MCF-7/TR and MDA-MB-231 cells treated with vehicle (−) and 50 or 100 μM of OLA for 48 h. One of three similar experiments is presented. **(B)** MCF-7/TR **(left panel)** and MDA-MB-231 **(right panel)** cells were treated with vehicle (−) and 50 or 100 μM of OLA for 48 h before lysis. Equal amounts of total cellular extracts were analyzed for PARP, caspase 3, and 8 protein expression from total cellular extracts. The histograms represent the mean ± SD of three separate experiments in which band intensities were evaluated in terms of optical density arbitrary units (OD) and expressed as a percentage of control which was assumed to be 100%. ^∗^*P* < 0.05 OLA treated vs vehicle-treated cells (−).

To corroborate the involvement of OLA in apoptosis, we first determined the fragmentation profile of genomic DNA (a typical biochemical hallmark of apoptotic cells death), in cells treated with OLA at 50 and 100 μM for 48 h, by DNA laddering. Chromosomal DNA extracted from MCF-7/TR and MDA-MB-231 cells revealed a marked DNA fragmentation consisting of multimers of 180–200 bp through agarose gel electrophoresis (Figure 8A).

**FIGURE 9:**
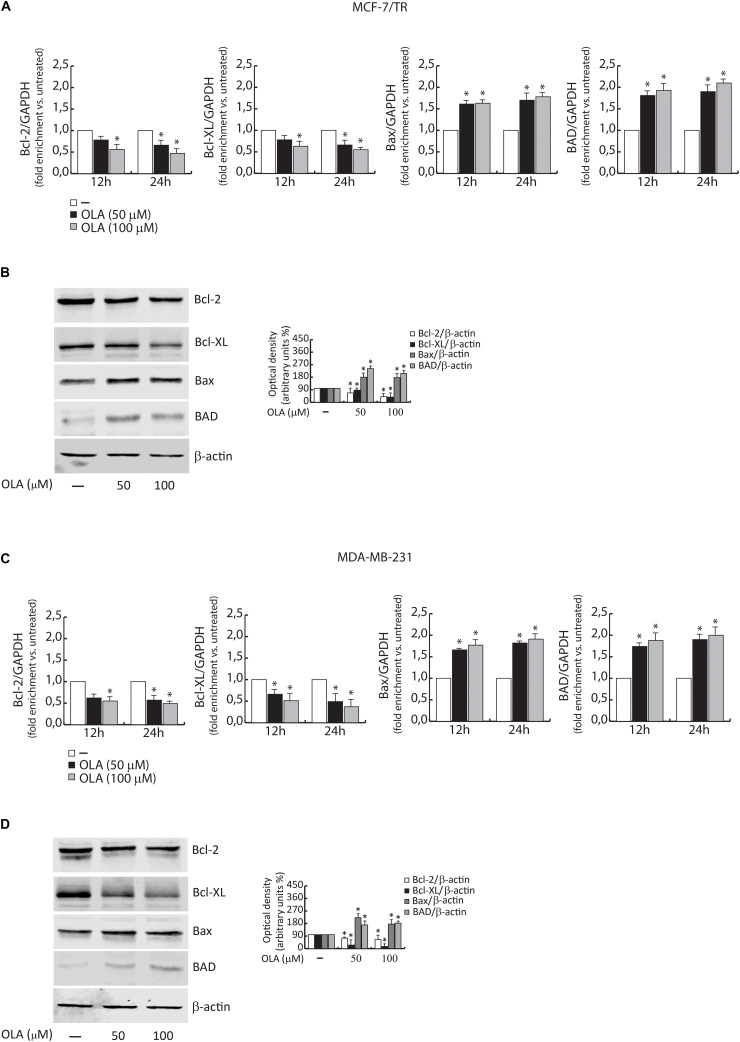
**(A)** MCF-7/TR and **(C)** MDA-MB-231 cells were treated with vehicle (−) and 50 or 100 μM of OLA compound for 12 and 24 h before lysis. Bcl-2/Bcl-XL and Bax/Bad mRNA content, evaluated by real-time RT-PCR. Each sample was normalized to its GAPDH mRNA content. The values represent the means ± S.D. of three different experiments, each performed in triplicate. **(B)** MCF-7/TR and **(D)** MDA-MB-231 cells were treated with vehicle (−) and 50 or 100 μM of OLA compound for 48 h before lysis. Immunoblots of Bcl-2/Bcl-XL and Bax/Bad were performed. B -actin was used as a loading control. The histograms represent the means ± SD of three separate experiments in which band intensities were evaluated in terms of optical density arbitrary units (OD) and expressed as a percentage of control which was assumed to be 100%. ^∗^*P* < 0.05 OLA treated vs vehicle-treated cells (−).

Next, the proteolysis of PARP, a known substrate of effector caspases, and the cleavage of caspase 3 and 8 by the immunoblotting analysis were evaluated. Results showed an increased level of the proteolytic form of PARP and caspase 3 and 8, in both BC cells treated for 48 h with OLA at 50 or 100 μM, compared to vehicle-treated cells (Figure 8B). Increased expression of Bax/Bad and a down-regulation of Bcl-2/Bcl-XL both at mRNA and protein levels in BCC treated with OLA as reported (Figures 9A–D) were found. Finally, the apoptosis effect induced by OLA in both BCCs is demonstrated by the increased level of the proteolytic form of PARP, as a pivot target that signals the presence of DNA damage and facilitates DNA repair, as well as marked DNA fragmentation. Besides, the activation of Bax/Bcl-2 signaling could suggest the activation of the intrinsic apoptotic pathway. In conclusion, all the results obtained evidence that the inhibition of cell cycle progression through apoptosis induction could explain one of the mechanisms by which OLA exhibits its antiproliferative effects in human BCCs.

It is important to highlight that the same results were obtained using OLA aliquots produced/extracted at different times by the integrated membrane process. This suggests the suitability/reproducibility of the process for the production of OLA as well as its unmodified anticancer properties and stability.

## Conclusion

In this work, an integrated membrane process was developed to produce OLA, an important phytotherapic compound generally present in olive oil.

The integrated membrane system was composed of an MBR, where the OLA was produced, and by a ME process, where the compound was extracted in a volatile green organic solvent. The substrate used in this reaction was oleuropein extracted from olive leaves by a sustainable method.

Under optimized conditions, we obtained an oleuropein conversion of about 93% for five consecutive reaction cycles and continuous extraction of OLA in the organic solvent (about 90%). The presence of all isomers of OLA, characterized by HPLC, H1-NMR, and LC-MS, confirmed the suitability of the integrated membrane process for the production/extraction of this compound since structural modification was observed.

The biosynthesized OLA was then tested as an antiproliferative drug toward two lines of BC cells: human ERα+ tamoxifen resistant (MCF-7/TR) and MDA-MB-231 (triple-negative). Results demonstrated that OLA caused a selective inhibition of cells growth just in BCC, without affecting the MCF-10A normal breast epithelial cells. It also induced G0/G1 cell cycle arrest, enhanced the CDK inhibitor p21Cip/WAF1, p27, p53 expression, and decreased cyclin D/E expression at both mRNA and protein levels. The increased cleavage/expression of proteins (PARP, caspase 3/8, Bax-Bad/Bcl-2-Bcl-XL) is typically observed during apoptotic phenomena and the significant DNA fragmentation also demonstrated the apoptotic activity of OLA in both the cell lines investigated.

This work proposed a productive strategy to obtain a compound with high therapeutic value by a sustainable technology, starting from waste/renewable material.

The mild production/extraction conditions used in the membrane process maintained the original properties of the phytotherapic compound. Based on the obtained results, OLA could be used for further studies as a potential tool that could be implemented for BC treatment, particularly to overcome the resistance of hormonal therapies.

## Data Availability Statement

The raw data supporting the conclusions of this article will be made available by the authors, without undue reservation.

## Author Contributions

RM developed the membrane bioreactor of the combined membrane system, also wrote the article, combined all the different part of the manuscript and the different activities. EP developed the membrane emulsification process for the extraction of the oleuropein aglycone, wrote and revised the manuscript. TP, FB, and LG wrote and revised the manuscript. MN, AP, and MD produced the substrate of the enzymatic reaction, they characterized the product obtained by membrane process and they wrote and revised the corresponding part of the article. RC and CM tested the anticancer properties of the oleuropein aglycone produced by the combined membrane process. PR analyzed and elaborated the data about the anticancer activity. MP coordinated the breast anticancer study, wrote the article and revised the part related to the mentioned activity in the manuscript. All authors contributed to the article and approved the submitted version.

## Conflict of Interest

The authors declare that the research was conducted in the absence of any commercial or financial relationships that could be construed as a potential conflict of interest.
