# Supplementary material for: Production of Plant-Derived Oleuropein Aglycone by a Combined Membrane Process and Evaluation of Its Breast Anticancer Properties
Source: Front Bioeng Biotechnol. 2020 Sep 29;8:908. doi: 10.3389/fbioe.2020.00908 (PMC7551858; doi:10.3389/fbioe.2020.00908)
Supplement: Supplementary file 1 [file Table_1.DOCX]

**Supporting information**

**Production of plant-derived oleuropein aglycone by a sustainable integrated membrane process and evaluation of its breast anticancer properties**

Rosalinda Mazzei^1^*, Emma Piacentini^1^, Monica Nardi^2^, Teresa Poerio^1^, Fabio Bazzarelli^1^, Antonio Procopio^2^, Maria Luisa Di Gioia^3^, Pietro Rizza^3^, Rosangela Ceraldi^3^, Catia Morelli^3^, Lidietta Giorno^1^, Michele Pellegrino^3^*

^1^ *Institute on Membrane Technology, National Research Council, ITM-CNR, via P. Bucci, 17/C, I-87030 Rende (Cosenza), Italy*

*^2^* *Department of Health Sciences, University Magna Græcia, Route Europa, I-88100, Germaneto Catanzaro, Italy*

^3^ *Department of Pharmacy, Health and Nutritional Sciences, University of Calabria, Rende, 87036 Cosenza, Italy*

^*^: corresponding authors; Tel.: +39 0984 492076; fax: +39 0984 402103;

e-mail addresses: [r.mazzei@itm.cnr.it](mailto:r.mazzei@itm.cnr.it) [pietrorizz@yahoo.it](mailto:pietrorizz@yahoo.it)

**
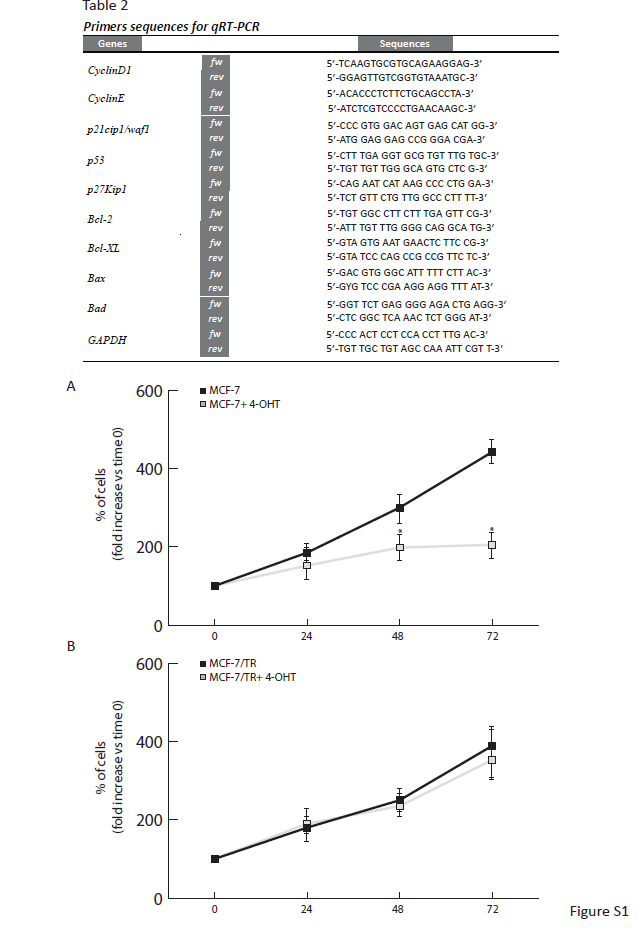
**

**Figure S1.** Growth curve response to tamoxifen of MCF-7/TR with respect to sensitive parental cells (MCF-7). MCF-7 (**A**) and MCF-7/TR (**B**) cells were starved ON and then switched to 5% PRF-CT and treated or not with 4-OHT 1mM for 24, 48 and 72 hours. At each time point, cells were tested on cell viability by using SRB assays. 4-OHT (1μM) treatment was refreshed every day to maintain constant levels in the medium. Data are reported as percentage of cell increase over time 0. Results are the mean ± SD of at least three independent experiments. *p < 0.05 *vs* untreated.

**Table S1** Primers sequences for qRT-PCR

**
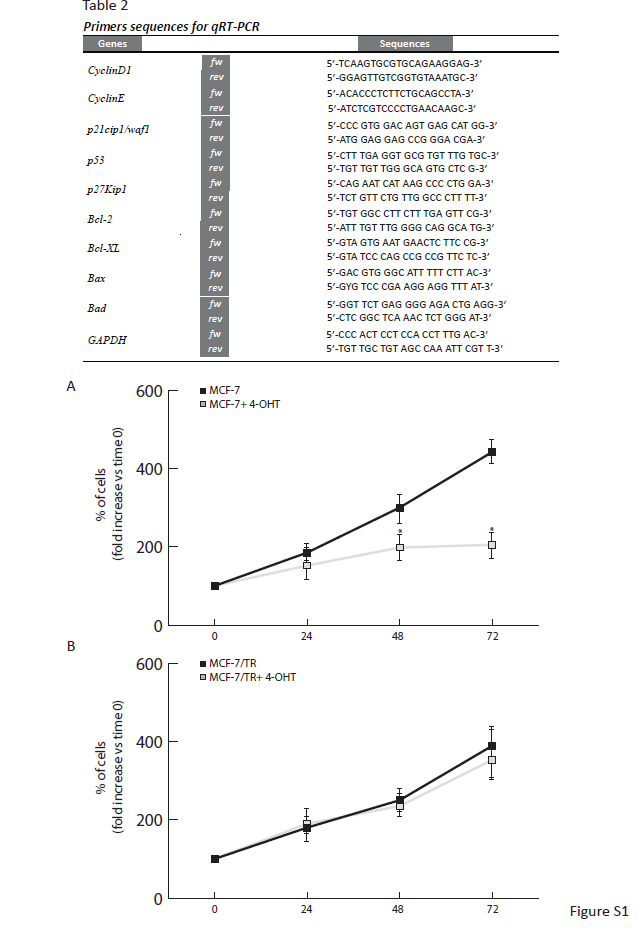
**

**Spectral data oleuropein**

The purity was determined by LC-QTOF-MS. ^1^H-NMR and ^13^C-NMR: Spectral data were in accordance with the literature (Goulas V. 2009) . LC-MS (m/z) calcd for C_25_H_32_O_13_ [M]^+^ 540.1843, measured 540.1828, found 563.1726 C_25_H_32_NaO_13_ [M+Na]^+^.

|  |  |  | |  | | |  | | | |  | |  | | |  | |  | | |  | | |  | | | |  | | |  | | | |  | |  | | | | | |  | |  | | |  | | |  | | | |  | | | |  | | |  | | |  | | | |  | | |  | | |  | | | | | |  | | | | | | | |  | | |  | | | |  | | |  | | | | |  | | | |  | | | | | | | | | | | | | | |
| --- | --- | --- | --- | --- | --- | --- | --- | --- | --- | --- | --- | --- | --- | --- | --- | --- | --- | --- | --- | --- | --- | --- | --- | --- | --- | --- | --- | --- | --- | --- | --- | --- | --- | --- | --- | --- | --- | --- | --- | --- | --- | --- | --- | --- | --- | --- | --- | --- | --- | --- | --- | --- | --- | --- | --- | --- | --- | --- | --- | --- | --- | --- | --- | --- | --- | --- | --- | --- | --- | --- | --- | --- | --- | --- | --- | --- | --- | --- | --- | --- | --- | --- | --- | --- | --- | --- | --- | --- | --- | --- | --- | --- | --- | --- | --- | --- | --- | --- | --- | --- | --- | --- | --- | --- | --- | --- | --- | --- | --- | --- | --- | --- | --- | --- | --- | --- | --- | --- | --- | --- | --- | --- |
| **Compound Label** | | | | | | | | | | **Name** | | | | | | | | | | | ***m/z*** | | | | | | | | | | | | **RT** | | | | | | | | **Algorithm** | | | | | | | | | | | | | | | **Mass** | | | | | | | | | | |  | | | | | | | | | | | |  | | | |  | | |  | | | |  | | | |  |  |  |  |  |  |  |  |  |  |  |  |  |  |  |  |  |  |  |  |  |  |  |  |  |  |  |  |  |
| oleuropein | | | | | | | | | | **oleuropein** | | | | | | | | | | | 563,1735 | | | | | | | | | | | | 3,856 | | | | | | | | Find By Formula | | | | | | | | | | | | | | | 540,1839 | | | | | | | | | | |  | | | | | | | | | | | |  | | | |  | | |  | | | |  | | | |  |  |  |  |  |  |  |  |  |  |  |  |  |  |  |  |  |  |  |  |  |  |  |  |  |  |  |  |  |
| 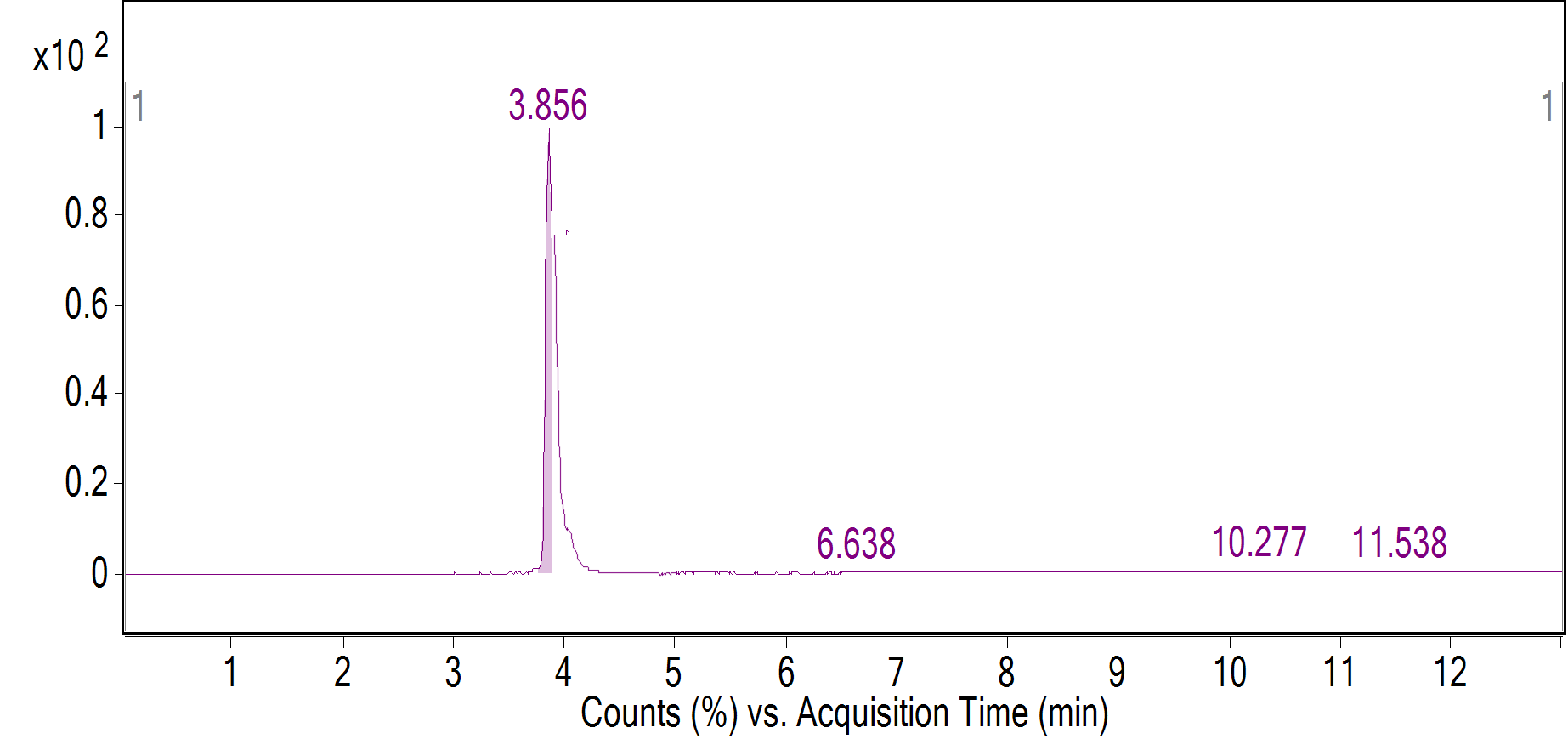   \| 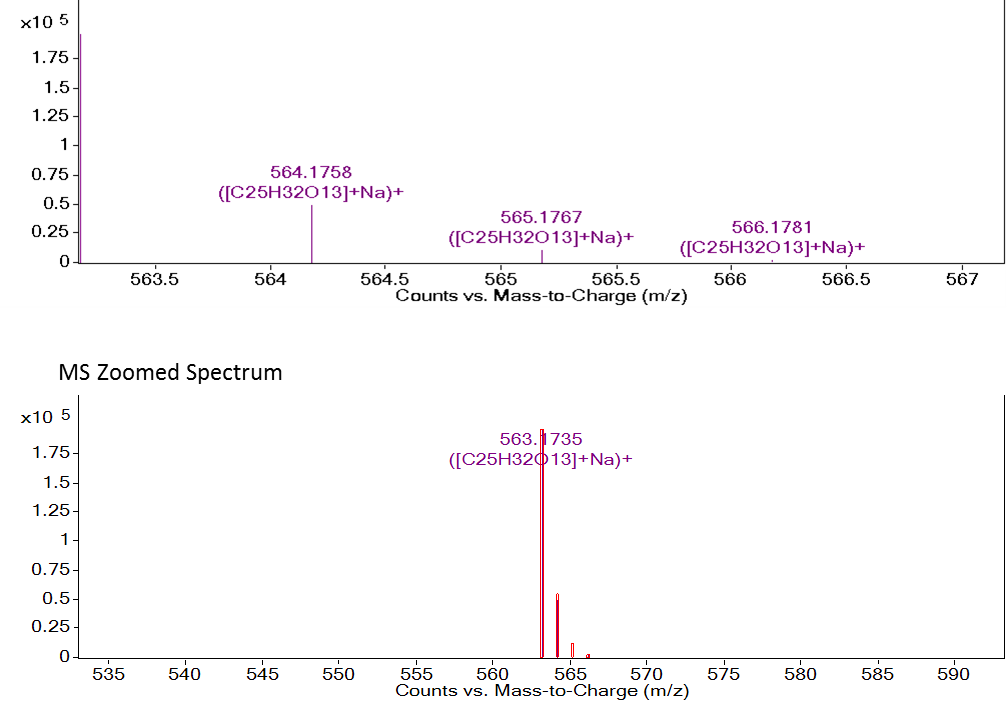 \| \| --- \| | | | | | | | | | | | | | | | | | | | | | | | | | | | | | | | | | | | | | | | | | | | | | | | | | | | | | | | | | | | | | | | | | | | | | | | | | | | | | | | | | | | | | | | | | | | | | | | | | | | | | | | | | | | | | | | | | | | | | | | | | |  |
| **MS Spectrum Peak List** | | | | | | | | | | | | | | | | | | |  | | |  | | | |  |  | | | | |  | | | |  | | | |  | | | | | |  | | |  | | | | |  | | |  | | | | | |  | | |  | | | |  | | | |  | | | |  | |  | | | | | | | |  | | | | |  | | | | | | |  | | | | | |  | | | | |  | | |  | | | |  | |  | | |
| ***m/z*** | | | | | | **z** | | | **Abund** | | | | | | **Formula** | | | | | | | | | | | | | | | | | | | | | **Ion** | | | |  | | | | | | | | | | | |  | |  | | | | | |  | | |  | | |  | | | |  | | |  | | | | |  | |  | | | | |  | | | | | | | |  | | | | |  | | | |  | | |  | | | | |  | | |  | | | |  |  |  |  |  |  |
| 563,1735 | | | | | | 1 | | | 196279,95 | | | | | | C25H32O13 | | | | | | | | | | | | | | | | | | | | | (M+Na)+ | | | |  | | | | | | | | | | | |  | |  | | | | | |  | | |  | | |  | | | |  | | |  | | | | |  | |  | | | | |  | | | | | | | |  | | | | |  | | | |  | | |  | | | | |  | | |  | | | |  |  |  |  |  |  |
| 564,1758 | | | | | | 1 | | | 49767,15 | | | | | | C25H32O13 | | | | | | | | | | | | | | | | | | | | | (M+Na)+ | | | |  | | | | | | | | | | | |  | |  | | | | | |  | | |  | | |  | | | |  | | |  | | | | |  | |  | | | | |  | | | | | | | |  | | | | |  | | | |  | | |  | | | | |  | | |  | | | |  |  |  |  |  |  |
| 565,1767 | | | | | | 1 | | | 11598,06 | | | | | | C25H32O13 | | | | | | | | | | | | | | | | | | | | | (M+Na)+ | | | |  | | | | | | | | | | | |  | |  | | | | | |  | | |  | | |  | | | |  | | |  | | | | |  | |  | | | | |  | | | | | | | |  | | | | |  | | | |  | | |  | | | | |  | | |  | | | |  |  |  |  |  |  |
| 566,1781 | | | | | | 1 | | | 1971,54 | | | | | | C25H32O13 | | | | | | | | | | | | | | | | | | | | | (M+Na)+ | | | |  | | | | | | | | | | | |  | |  | | | | | |  | | |  | | |  | | | |  | | |  | | | | |  | |  | | | | |  | | | | | | | |  | | | | |  | | | |  | | |  | | | | |  | | |  | | | |  |  |  |  |  |  |
| 567,18 | | | | | | 1 | | | 324,16 | | | | | | C25H32O13 | | | | | | | | | | | | | | | | | | | | | (M+Na)+ | | | |  | | | | | | | | | | | |  | |  | | | | | |  | | |  | | |  | | | |  | | |  | | | | |  | |  | | | | |  | | | | | | | |  | | | | |  | | | |  | | |  | | | | |  | | |  | | | |  |  |  |  |  |  |
|  |  | |  | |  | | |  | | | |  | |  | | |  | | |  | | |  | |  | | | |  |  | | | |  | | | |  |  | | |  | |  | | |  | | |  | | |  | | | | |  | | |  | | |  | | | |  | | |  | | | | |  |  | | | | |  | |  | | |  | | | |  | | | |  | |  | | | |  | |  | | | |  | |  | | |  | | |  |  | | |  | |  | |

**Figure S2.** Oleuropein Data spectra obtained by LC-MS

**Spectral data of OLA (3,4-DHPEA-EA)**

H^1^-NMR (CDCl_3_) δ 1.41 [d, 3H, CH_3_ (E), J= 6.64 Hz], 1.41 [d, 3H, CH_3_ (C), J= 6.76 Hz], 2.13-2.28 [m, 2H, H (E)], 2.50-2.63 [m, 2H, H (C)], 2.78-2.85 [t, 2H, J= 5.77 Hz], 2.87 (t, 2H, H_7_, J= 5.85 Hz), 3.36 [m, 1H, H4 (C)], 3.74 [s, 3H, OCH_3_ (E)], 3.77 [s, 3H, OCH_3_ (C)], 4.10-4.50 [m, 6H, H (E), H (C), 2H (E), 2H (C)], 6.62[m, 2H, H_aromatic_ (C)], 6.75 [m, 2H, H_aromatic_ (E)], 7.26 [s, 2H, H_aromatic_ (C, E)], 7.58 [s, 1H, H(E)], 9.52 [s, 1H, H(C)], 9.60 [s, 1H, H (C)], 9.80 [s, 1H, H (B)]


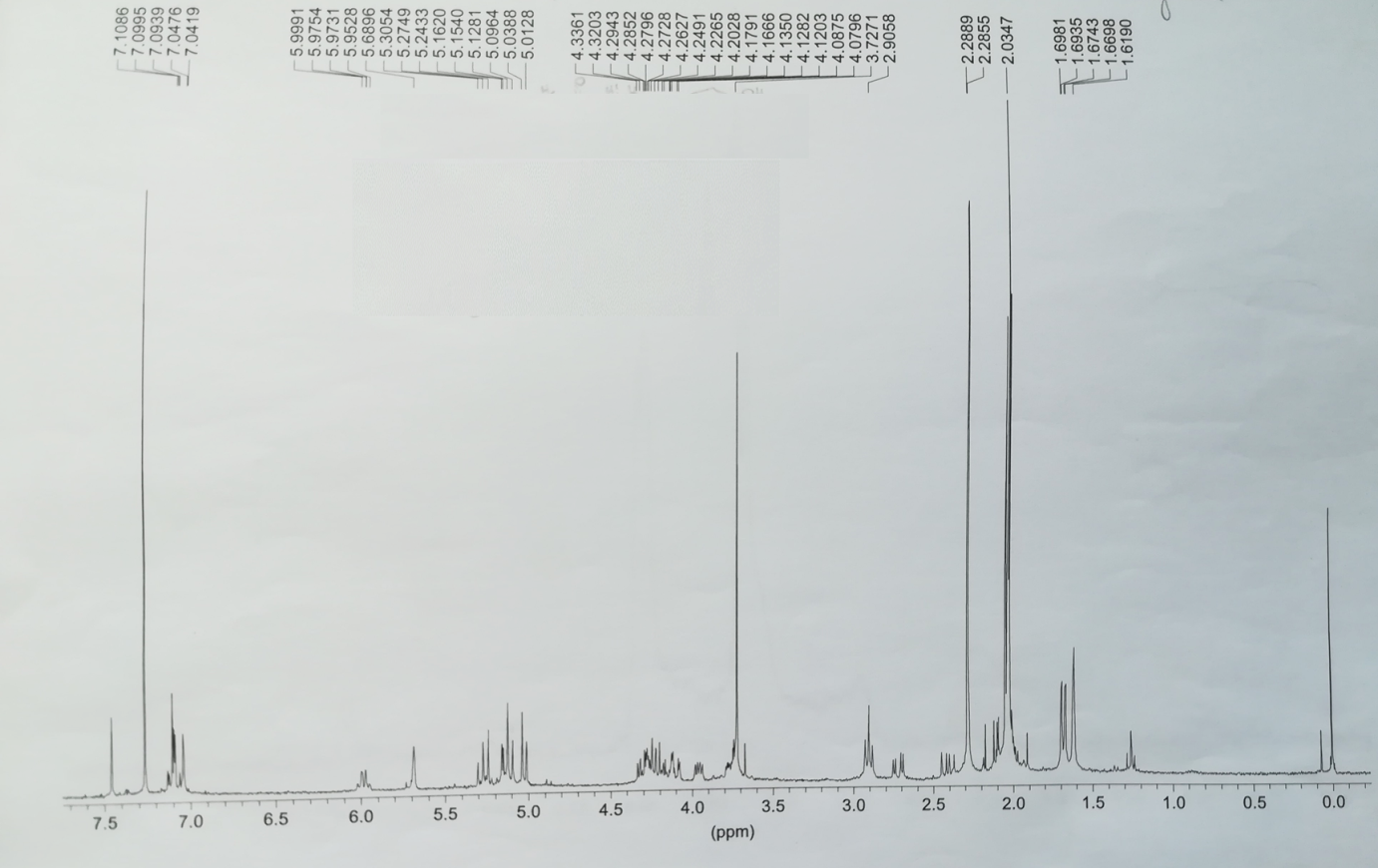


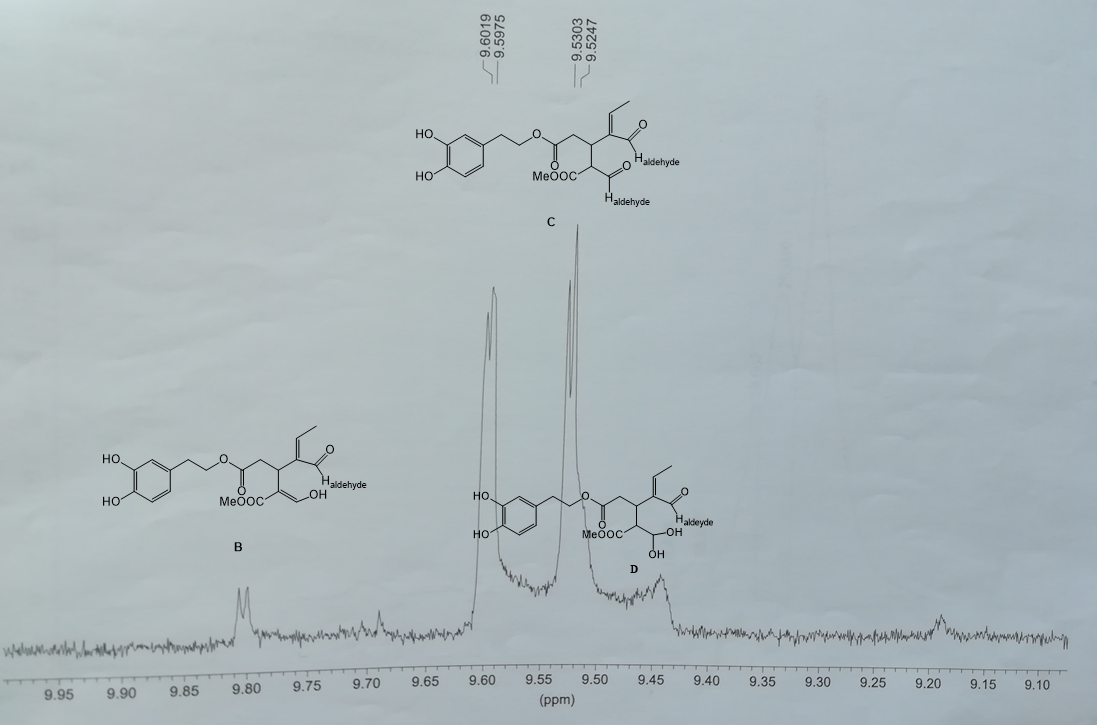


**Figure S3.** ^1^H-NMR Aglicone Oleuropeina (**3,4-DHPEA-EA)**


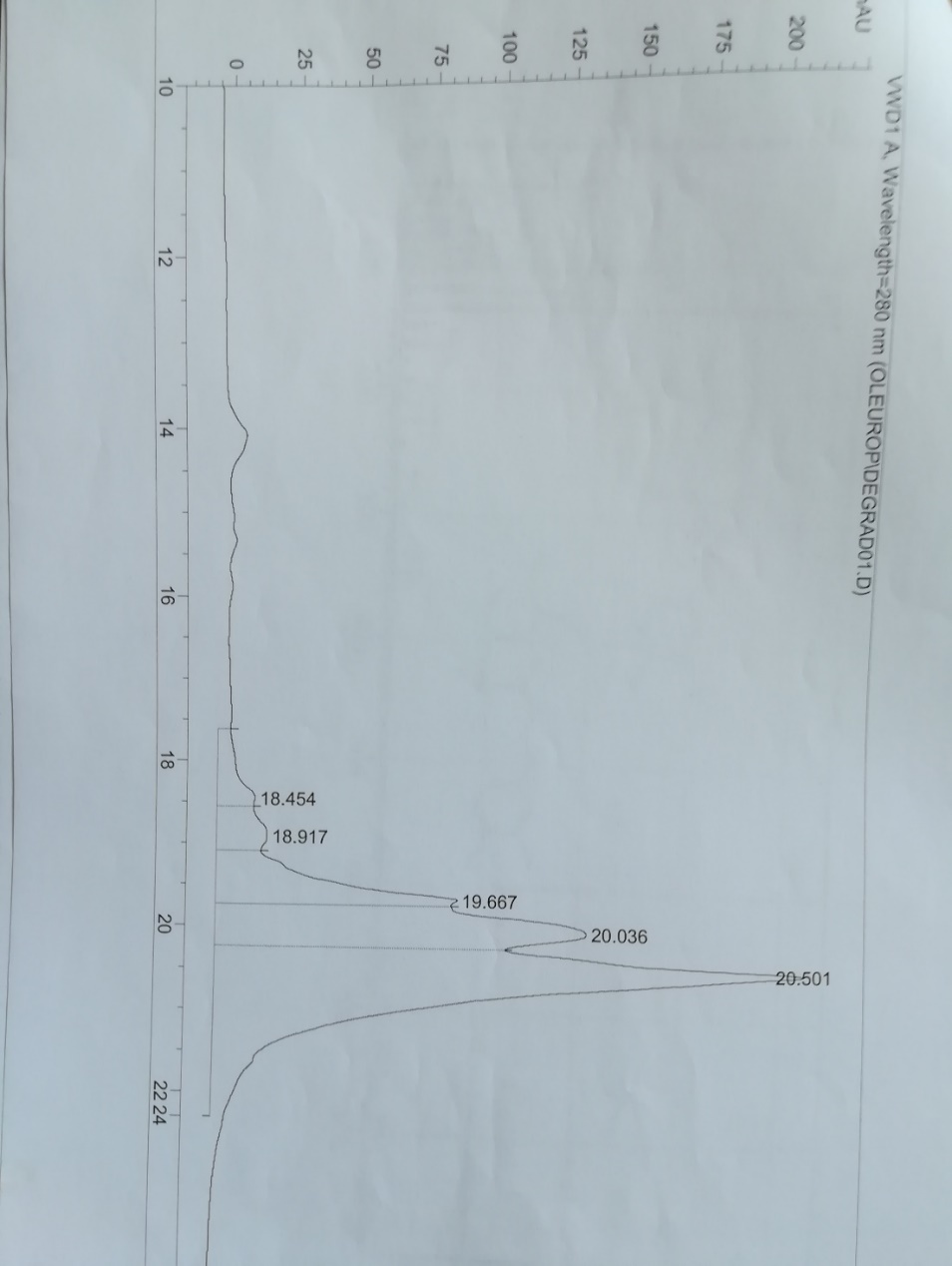


**Fig. S4.** HPLC of Aglicone Oleuropeina (**3,4-DHPEA-EA)**

LC-MS (m/z) calcd for C_19_H_22_O_8_ [M]^+^ 378.1315, measured 378.1309, found 401.1200 C_19_H_22_NaO_8_ [M+Na]^+^.

**HPLC-ESI-QTOF-MS** tr = 4.364 min m/z 401. 1172 [M + Na^+^].

|  |  |  |  |  |  |  |  |  |  |  |  |  | |  |  |  |  |  |  |  |  |  |  |  |  |  |  |  |
| --- | --- | --- | --- | --- | --- | --- | --- | --- | --- | --- | --- | --- | --- | --- | --- | --- | --- | --- | --- | --- | --- | --- | --- | --- | --- | --- | --- | --- |
| **Compound Label** | | | | | | **Name** | | | | | | | ***m/z*** | | | | **RT** | | **Algorithm** | | | | | | **Mass** | | | |
| Oleuropein Aglycone | | | | | | **Oleuropein Aglycone** | | | | | | | 401,1172 | | | | 4,364 | | Find By Formula | | | | | | 378,1278 | | | |
|  |  |  |  |  |  |  |  |  |  |  |  |  | |  |  |  |  |  |  |  |  |  |  |  |  |  |  |  |
|    \|  \| \| --- \| |  |  |  |  |  |  |  |  |  |  |  |  | |  |  |  |  |  |  |  |  |  |  |  |  |  |  |  |
| MS Spectrum | | |  |  |  |  |  |  |  |  |  |  | |  |  |  |  |  |  |  |  |  |  |  |  |  |  |  |
| \|  \| \| --- \| |  |  |  |  |  |  |  |  |  |  |  |  | |  |  |  |  |  |  |  |  |  |  |  |  |  |  |  |
| MS Zoomed Spectrum | | | |  |  |  |  |  |  |  |  |  | |  |  |  |  |  |  |  |  |  |  |  |  |  |  |  |
|    \|  \| \| --- \| |  |  |  |  |  |  |  |  |  |  |  |  | |  |  |  |  |  |  |  |  |  |  |  |  |  |  |  |
| **MS Spectrum Peak List** | | | | | | | |  |  |  |  | |  |  |  |  |  |  |  |  |  |  |  |  |  |  |  |  |
| ***m/z*** | | | **z** | **Abund** | | | **Formula** | | | | | | | **Ion** | | |  |  |  |  |  |  |  |  |  |  |  |  |
| 401,1172 | | | 1 | 62217,47 | | | C19H22O8 | | | | | | | (M+Na)+ | | |  |  |  |  |  |  |  |  |  |  |  |  |
| 402,1202 | | | 1 | 11596,97 | | | C19H22O8 | | | | | | | (M+Na)+ | | |  |  |  |  |  |  |  |  |  |  |  |  |
| 403,1188 | | | 1 | 2325,22 | | | C19H22O8 | | | | | | | (M+Na)+ | | |  |  |  |  |  |  |  |  |  |  |  |  |
| 404,1184 | | | 1 | 321,16 | | | C19H22O8 | | | | | | | (M+Na)+ | | |  |  |  |  |  |  |  |  |  |  |  |  |

**Fig. S5** Spectral data obtained by LCMS of oleuropein aglycone


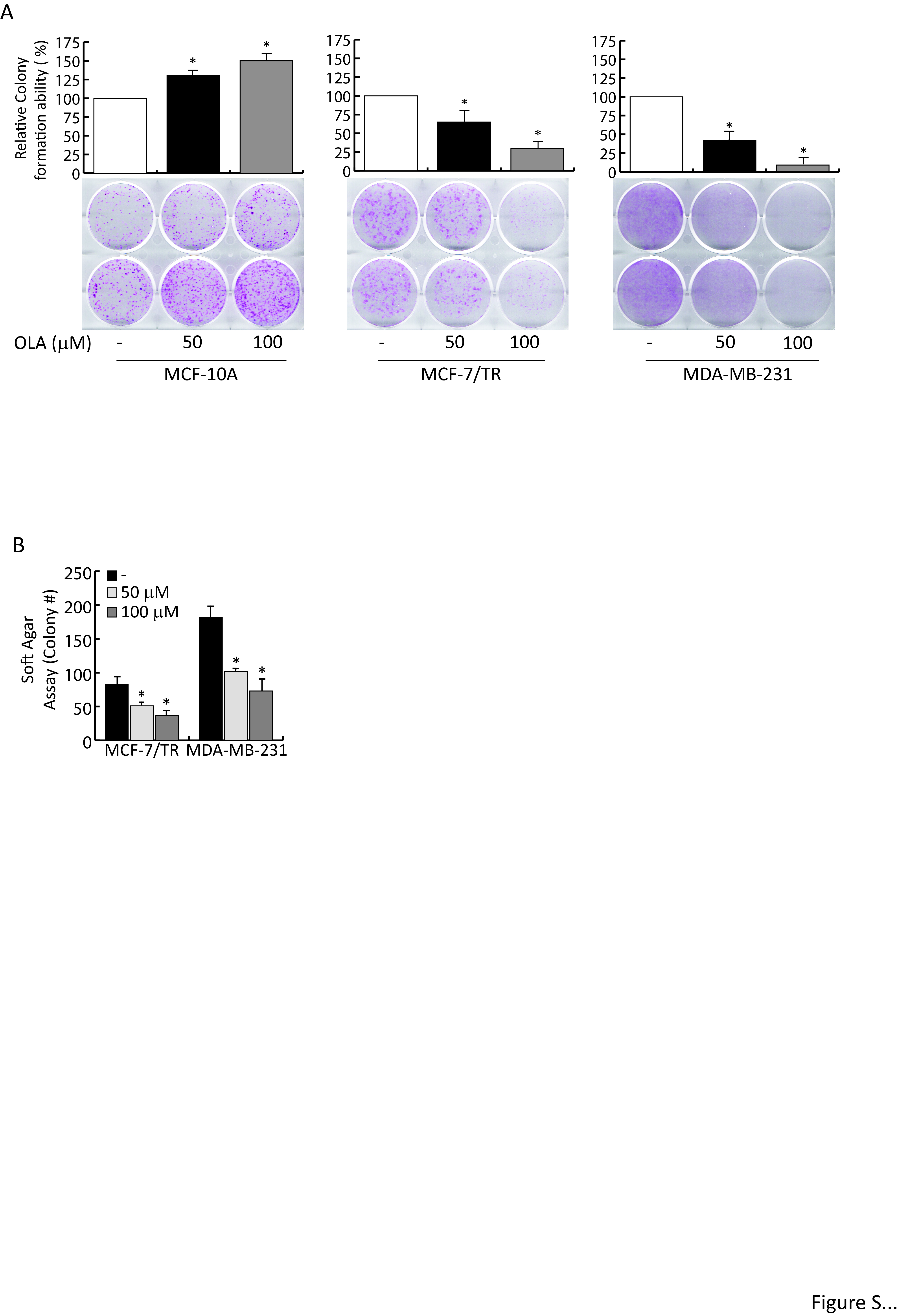


**Fig. 6S** Antiproliferative effect on BCC induced by OLA in anchorage dependent and independent conditions (A) Clonogenic assay. Cells were plated in duplicate in 6 well plates and treated vehicle (-) or OLA 50 and 100 μM. After 2 weeks, colonies were stained with crystal violet and counted. Upper panel, the histograms represent the mean ± SD of three separate experiments in which band intensities were evaluated in terms of optical density arbitrary units and expressed as percentage of control which was assumed to be 100%. *p < 0.05 compared to vehicle (-) treated cells. (B) Soft-agar growth assays. MCF-7/TR and MDA-MB-231 cells were plated in 0.35% agarose and treated with vehicle (-) and 50 or 100 μM of OLA compound, as indicated. After 14 days of growth, colonies >50 μm diameter were quantified. The values represent the mean ± SD of two different experiments, each performed in triplicate.

References

Goulas V., Exarchou V., Troganis A.N., Psomiadou E., Fotsis T., Briasoulis E., Gerothanass I. P., (2009). Phytochemicals in Olive-Leaf Extract and Their Antiproliferative Activity Against Cancer and Endothelial Cells. Mol. Nutr. Food Res., 53, 600-608.
